# Pneumothorax Ex Vacuo

**DOI:** 10.1002/jgf2.70137

**Published:** 2026-05-28

**Authors:** Yusuke Ito

**Affiliations:** ^1^ Department of Family Practice Azusawa Hospital Tokyo Japan

**Keywords:** lung neoplasms, pleural effusion, pneumothorax, thoracentesis

## Abstract

An 84‐year‐old woman with pleural effusion developed anterior chest pain during thoracentesis, and post‐procedural chest radiography revealed hydropneumothorax, initially suggesting iatrogenic pneumothorax. However, comparison of pre‐ and post‐procedure imaging showed ipsilateral hemithoracic volume loss with tracheal deviation toward the procedure side, supporting pneumothorax *ex vacuo*, managed conservatively without intervention.

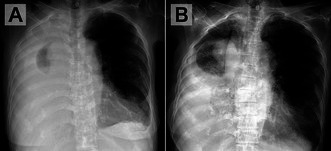

An 84‐year‐old woman was admitted for evaluation of two months of general fatigue and a right‐sided pleural effusion (PE) (Figure 1A). On admission, mild hypoxemia with an oxygen saturation of 93% on ambient air was observed. During thoracentesis, no air aspiration or changes in vital signs was observed; however, the patient developed right anterior chest pain, prompting discontinuation of the procedure after 900 mL drainage. A post‐thoracentesis chest radiograph demonstrated a hydropneumothorax (Figure 1B).

**FIGURE 1:**
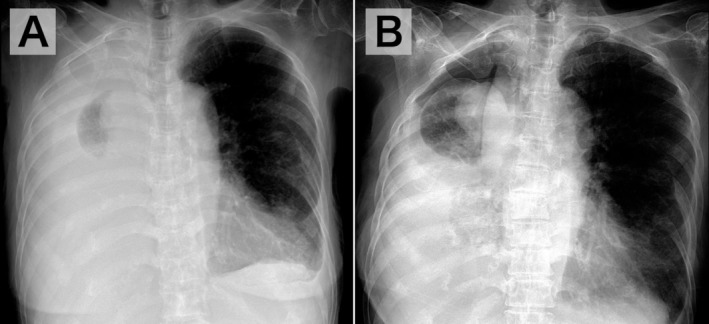
Chest radiographs obtained before (A) and after (B) thoracentesis. (A) Posteroanterior view in the standing position. (B) Anteroposterior view in the seated position. Compared with the pre‐thoracentesis image, the post‐thoracentesis radiograph shows a reduction in right thoracic volume and bronchial deviation toward the procedural side.

Initially, iatrogenic pneumothorax was suspected. However, comparison with pre‐thoracentesis imaging revealed bronchial deviation toward the procedural side and a loss of thoracic volume. Computed tomography showed a right hilar tumor obstructing the bronchus without visceral pleural thickening (Figure 2A,B), and follow‐up imaging demonstrated reaccumulation of the PE without a change in lung shape (Figure 2C,D), consistent with pneumothorax ex vacuo.

**FIGURE 2:**
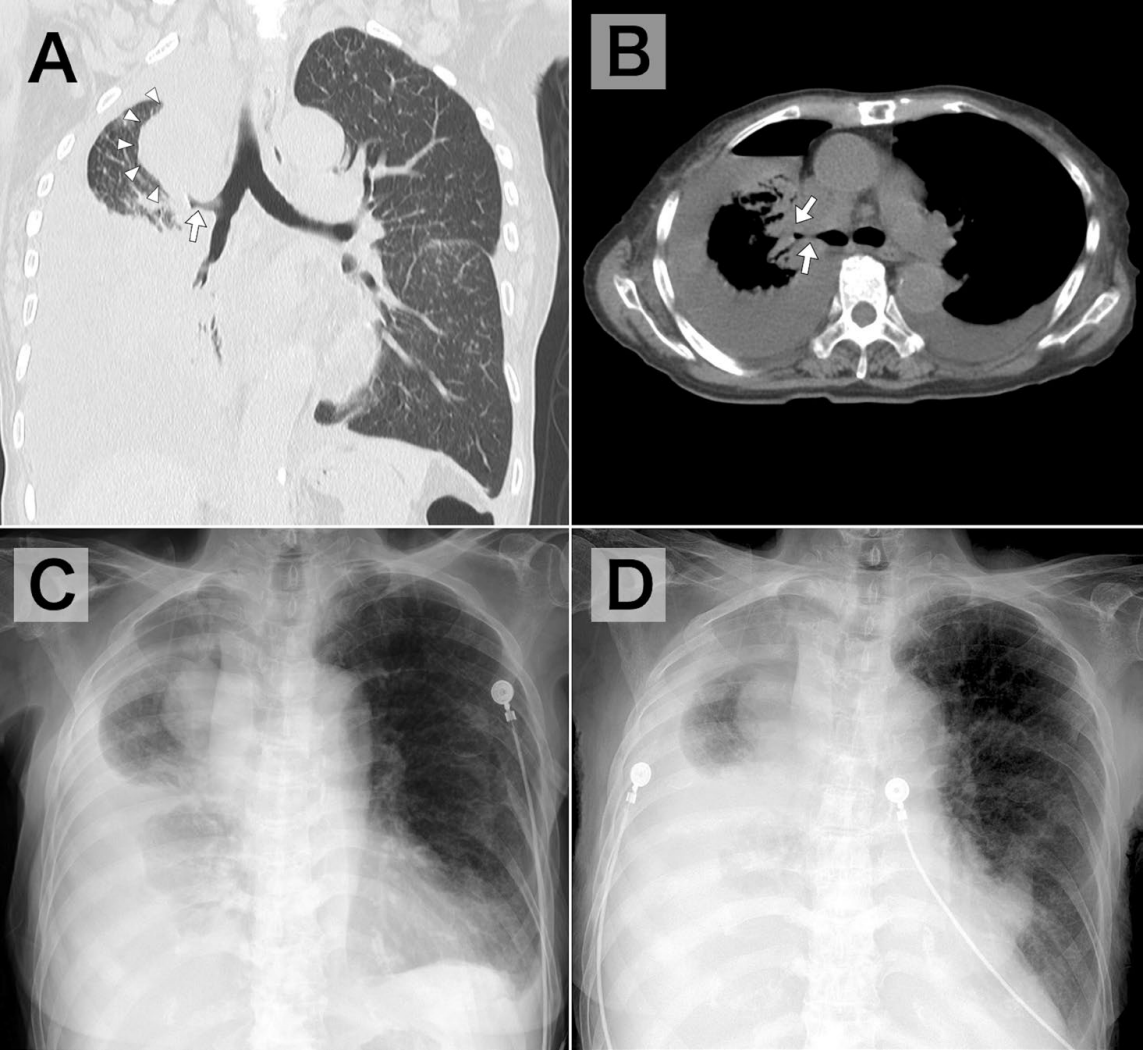
(A) Computed tomography obtained after thoracentesis, revealing a right hilar tumor (white arrowheads) obstructing the right bronchus (white arrow). (B) Axial mediastinal‐window view demonstrating obstruction of the right bronchus (white arrows). (C, D) Follow‐up chest radiographs (anteroposterior view, seated position) obtained 2 and 5 days after thoracentesis, respectively, showing reaccumulation of pleural effusion without lung shape change.

The PE was exudative, and cytology was consistent with lung adenocarcinoma, leading to a diagnosis of non‐expandable lung (NEL) due to lung cancer. Further investigations revealed bone and brain metastases. She received palliative care and died one month later.

The presence of air in the pleural space on a post‐thoracentesis chest radiograph does not necessarily indicate an iatrogenic pneumothorax. Clinicians should consider the following three conditions [1, 2]. The first is iatrogenic pneumothorax, the most concerning entity, requiring chest tube insertion in approximately one‐third of cases [2]. The second is atmospheric air entrainment via the incision site, generally requiring no intervention [1, 2]. Finally, pneumothorax ex vacuo—a condition often challenging to distinguish from iatrogenic pneumothorax—develops after PE drainage in NEL, characterized by impaired lung re‐expansion due to bronchial obstruction or visceral pleural thickening [1, 2].

NEL mainly occurs as a complication of various pleural pathologies, affecting 3%–34% of malignant PEs [3]. NEL is classified as lung entrapment or trapped lung, depending on whether active pleural disease is present [3]. Lung entrapment arises in the setting of active visceral pleuritis with exudative PE [3]. Trapped lung results from defective healing of pleuritis, leaving a fibrous visceral pleural peel, with paucicellular transudate or protein‐discordant exudate PE [3, 4]. In trapped lung, visceral pleural thickening reflecting fibrosis may be seen on air‐contrast computed tomography [4, 5]. In this case, however, it was not evident, possibly because air‐contrast computed tomography was not performed or because the malignant pleuritis remained active. In addition, bronchial obstruction caused by the tumor may also have contributed to the NEL.

Diagnosis of pneumothorax ex vacuo requires identification of NEL. When feasible, pleural manometry during thoracentesis showing high pleural elastance suggests its presence [4].

Without pleural manometry, the clinical findings of iatrogenic pneumothorax and pneumothorax ex vacuo may overlap, making differentiation challenging. In pneumothorax ex vacuo, PE drainage may cause anterior “pressure‐like” chest pain radiating to the neck due to a decline in pleural pressure, commonly lasting for several minutes [4, 5]. Although air aspiration during thoracentesis has traditionally been considered a hallmark of iatrogenic pneumothorax [1], it can also occur in pneumothorax ex vacuo under negative‐pressure suction [5].

As in this case, comparing pre‐ and post‐thoracentesis chest radiographs can help differentiate iatrogenic pneumothorax from pneumothorax ex vacuo, demonstrating ipsilateral hemithorax volume loss (elevated diaphragm, narrowed intercostal spaces, and mediastinal shift toward the procedural side) with follow‐up imaging showing an unchanged lung shape and PE reaccumulation [1, 5]. Although these findings may not always be as clearly evident as in this case, their recognition remains clinically useful.

Pneumothorax ex vacuo generally requires no intervention, as it reflects re‐equilibration of intra‐ and extra‐pulmonary pressures; management should focus on the underlying NEL [5].

In conclusion, this case demonstrated distinctive radiographic findings of pneumothorax ex vacuo that became evident after thoracentesis, namely ipsilateral hemithorax volume loss and bronchial deviation reflecting reduced pleural pressure. Careful comparison of chest radiographs obtained before and after thoracentesis may facilitate accurate diagnosis and help avoid unnecessary invasive treatment.

## Author Contributions

**Yusuke Ito:** conceptualization, writing – original draft, writing – review and editing.

## Ethics Statement

Ethics approval was waived by the institutional review board because of the design of this study.

## Consent

Written informed consent was obtained from the patient's husband because the patient was deceased.

## Conflicts of Interest

The author declares no conflicts of interest.

## Data Availability

The data that support the findings of this study are available upon request from the corresponding author. The data are not publicly available due to privacy or ethical restrictions.
